# Quantitative CT: Associations between Emphysema, Airway Wall Thickness and Body Composition in COPD

**DOI:** 10.1155/2011/419328

**Published:** 2011-01-16

**Authors:** Erica P. A. Rutten, Thomas B. Grydeland, Sreekumar G. Pillai, Scott Wagers, Asger Dirksen, Harvey O. Coxson, Amund Gulsvik, Emiel F. M. Wouters, Per S. Bakke

**Affiliations:** ^1^Center of Expertise for Chronic Organ Failure (Ciro), 6085 NM Horn, The Netherlands; ^2^Department of Respiratory Medicine, Maastricht University Medical Center+ (MUMC+), P.O. Box 616, 6200 MD Maastricht, The Netherlands; ^3^Department of Thoracic Medicine, Haukeland University Hospital, 5021 Bergen, Norway; ^4^Section of Thoracic Medicine, Institute of Medicine, University of Bergen, 5021 Bergen, Norway; ^5^GlaxoSmithKline, Research Triangle Park, 27709 NC, USA; ^6^Department of Respiratory Medicine, Gentofte Hospital, University of Copenhagen, 27709 Copenhagen, Denmark; ^7^Department of Radiology, Vancouver General Hospital and the James Hogg iCAPTURE Centre for Cardiovascular and Pulmonary Research, University of British Columbia, Vancouver, BC, Canada V5Z 1M9

## Abstract

The objective of the present study was to determine the association between CT phenotypes—emphysema by low attenuation area and bronchitis by airway wall thickness—and body composition parameters in a large cohort of subjects with and without COPD. In 452 COPD subjects and 459 subjects without COPD, CT scans were performed to determine emphysema (%LAA), airway wall thickness (AWT-Pi10), and lung mass. Muscle wasting based on FFMI was assessed by bioelectrical impedance. In both the men and women with COPD, FFMI was negatively associated with %LAA. FMI was positively associated with AWT-Pi10 in both subjects with and without COPD. Among the subjects with muscle wasting, the percentage emphysema was high, but the predictive value was moderate. In conclusion, the present study strengthens the hypothesis that the subgroup of COPD cases with muscle wasting have emphysema. Airway wall thickness is positively associated with fat mass index in both subjects with and without COPD.

## 1. Introduction

Chronic obstructive pulmonary disease (COPD) is, besides a respiratory disorder, recognized as a systemic disease with extrapulmonary manifestations. In the past, the terms “pink puffer” and “blue bloater” were used to describe “phenotypes” of COPD, implying that the disease processes extended beyond the lung [1]. The pink puffers were characterized as having emphysematous lungs and a thin body habitus, while the blue bloaters were considered as being obese and having central cyanosis with little or no emphysema, often considered as the chronic bronchitis subtype of COPD. 

Current techniques utilizing quantitative computed tomography of thorax (CT) enables us to better characterize these phenotypes. It has recently been shown that the extent of emphysema measured by percentage of low attenuation areas (%LAA) is inversely associated with body mass index (BMI: body weight/height^2^) [2]. Another recent paper confirmed these data by showing an inverse correlation between the %LAA and both fat free-mass index (FFMI, a marker for skeletal muscle mass: fat free mass (FFM)/height^2^) and fat mass index (FMI, fat mass (FM)/height^2^) in subjects with COPD [3]. Both studies [2, 3] were restricted to heavy smoking men. 

Airway wall thickness, also derived from the CT scan, has recently been associated with symptoms of chronic bronchitis [4]. The relation between airway wall thickness and body composition is however controversial. Studies have shown both no [2] and positive [5] correlations between airway wall percent and BMI. As it is recently shown that airway wall thickening and emphysema make independent contributions to symptoms indicative of COPD [6], it is of relevance to test the discriminative capacity of the CT phenotypes for markers of extrapulmonary features of COPD like body composition. 

Based on the above-mentioned findings, we hypothesized that presence of significant emphysematous lesions on a thoracic CT scan is negatively associated with quantitative measures of systemic soft tissue, namely, total body fat-free mass. In addition, we hypothesized that airway wall thickness is positively associated with BMI and FMI in patients with COPD, reflecting the bronchitis phenotype. For this purpose, we examined a large cohort of subjects with COPD and a cohort of subjects without COPD, both men and women. 

## 2. Methods and Materials

Details of the population characteristics are given elsewhere [7–9]. Briefly, the study comprised 452 COPD cases and 459 subjects without COPD. Enrollment criteria were self-reported Caucasian; age >40; current or former smoker with ≥2.5 pack years of smoking history; no *α*_1_-antitrypsin deficiency. The medical ethical committee approved the study and written informed consent was obtained from all subjects. Subjects underwent spirometry according to ATS standards [4], using a Vitalograph 2160 Spirometer before and after bronchodilation with 400 *μ*g of salbutamol. The diagnostic criteria for COPD cases was postbronchodilator FEV1/FVC ratio less than 0.70 and FEV1 less than 80% of the predicted. Subjects without COPD had a postbronchodilator FEV1/FVC ratio greater than 0.70 and FEV1 greater than 80% of predicted [10]. Subjects were assessed at least 6 weeks after any respiratory infection, but were not asked to withhold regular medication. 

Subsequent to the assessment of body height and weight, whole body impedance was measured using the bio-electrical impedance method (Bodystat) after overnight fast. For the calculation of the FFM, a formula for elderly was used for the control subjects [11], while a COPD-specific formula was used for the COPD subjects [12]. FM was calculated by weight minus FFM, FFMI, and FMI was calculated by respectively FFM and FM divided by heigth^2^. Muscle wasting was defined by the cut-offs of Vestbo et al. [13]: FFMI < 17.1 kg/m^2^ for men and <14.6 kg/m^2^ for women. 

CT scans of thorax were acquired using a GE lightspeed Ultra CT scanner (120 kVp, 200 mA; GE Healthcare, Milwaukee, WI, USA), at suspended full inspiration using 1 mm slice thickness at 20 mm intervals and reconstructed using a high-resolution algorithm (Bone). On average there were 13.4 ± 1.6 CT images per subject. The procedures for quantitative analyses are previously described [14]. The extent of emphysema was assessed using custom software (EmphylxJ). A subject was considered to have emphysema if the percentage of lung voxels with X-ray attenuation values were less than −950 HU (%LAA) > 5%  [15]. The standardized airway wall thickening at an internal perimeter of 10 mm (AWT-Pi10) was calculated separately for each subject. To reduce technical errors associated with very small airways, only airways with an internal perimeter larger than 6 mm were included. Lung mass was calculated by multiplying CT calculated lung density (g/ml) by CT calculated lung volume (ml). The CT image analyses were performed at the James Hogg iCAPTURE Centre (Vancouver, BC, Canada). Further details of the CT analyses can be found elsewhere [4, 10].

### 2.1. Statistics

All data were expressed as mean ± SD, checked for normality, and if necessary log transformed (%LAA). Group comparison of continuous variables was tested using the analysis of variance (ANOVA) test and the post hoc LSD or the Dunnett-T3 test. Spearman correlation coefficient was calculated to test associations between CT variables and body composition parameters. Subsequently, multivariate linear regression analyses with log(%LAA) and AWT-Pi10 as dependent variables were performed to investigate their relationship to the body composition variables. Covariables in these analyses were age, pack years, and lung function. The prevalence of high emphysema scores after stratification for muscle wasting was tested by *γ* ^2^ test. As FFMI was independently associated with %LAA in the subjects with COPD, it is of relevance to test the discriminative capacity of FFMI for %LAA. For this purpose, the area under the curve (AUC) was calculated by using the receiving operating characteristic (ROC) curve. An AUC of 0.7–0.8 is considered to represent reasonable discrimination and an AUC > 0.8 suggest good discrimination [16]. Analyses were performed using Statistical Package for the Social Sciences (SPSS) version 17.0 for Windows. A *P*  value < .05 was considered statistically significant. 

## 3. Results

Characteristics of the study participants are presented in Table 1. Subjects without COPD were younger and had higher FEV_1_, FEV_1_/FVC, lung mass, weight, and FFMI, but lower amount of pack years smoked, AWT-Pi10, and %LAA than subjects with COPD. Within the groups, men had higher weight, FFMI, lung mass, amount of pack years smoked and AWT-Pi10 and lower FMI than women. Only in women with COPD, age and %LAA were lower compared to men with COPD. 

In the male and female COPD cases, %LAA was inversely correlated with BMI, FFMI, and FMI (Table 2). AWT-Pi10 was positively correlated with BMI, FFMI, and FMI in both subjects with and without COPD. In addition, only in the subjects with COPD, lung mass was significantly correlated with %LAA (*r* = −0.26, *P* < .01), and the correlation remained after correction for FFMI (*r* = −0.23, *P* < .01). 

In the subjects without COPD, the multivariate regression analysis revealed that FMI was significantly associated to %LAA, while both FMI and FFMI were significantly associated with AWT-Pi10 (Table 3). In the subjects with COPD, FFMI was a negative covariate for %LAA, while FMI was a positive covariate for AWT-Pi10. The associations did not differ between men and women (data not shown). 

**Figure 1:**
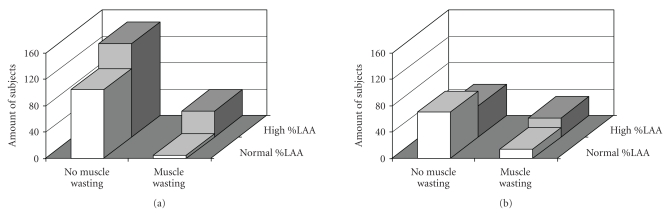
The amount of men (a) and women (b) with normal or high %LAA in relation to muscle wasting.

**Figure 2:**
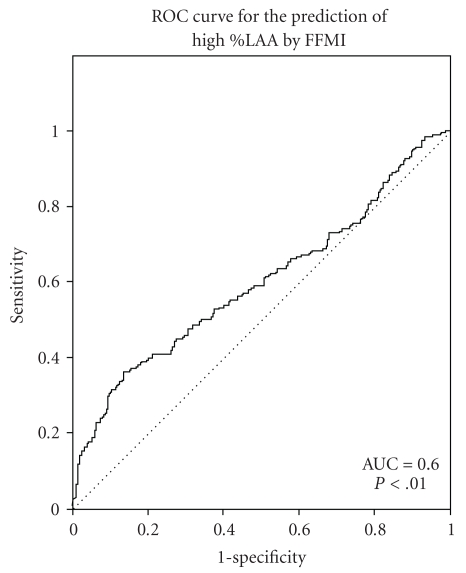
Receiving operating characteristics for FFMI in the subjects with COPD.

Of the male COPD cases, 15.5% had muscle wasting; of these, 88.9% had high emphysema scores (Figure 1). In women, about 27% of the COPD cases had muscle wasting; 67.4% of them had high emphysema scores. The other way around, of all the men with high emphysema scores (62.5%), only 22% of them had muscle wasting. In line, of the 47.5% of the women with high emphysema scores, about 28% of them had muscle wasting. Discriminating capacity of FFMI to predict high %LAA is presented in Figure 2. The AUC for FFMI in predicting high %LAA was 0.60 (95% confidence interval (CI): 0.55–0.65; sensitivity: 26.7; specificity: 90.2), indicating a moderate fit.

## 4. Discussion

The results of the present study reveal that fat free mass index decreases as the level of emphysema increases in both men and women with COPD. This relationship was independent of age, pack years, FEV1 (%pred), and airway wall thickness. However, muscle wasting is only moderate discriminative for the presence of emphysema. Furthermore, airway wall thickness was positively associated with fat mass index both in subjects with and without COPD. 

An inverse association of %LAA to FFMI and/or FMI has previously been observed in men with an extreme smoking history [2, 3]. We have extended this knowledge by showing that the association applies to both genders and exists also in subjects with a far less smoking burden (e.g., mean pack years = 65 in [3] versus 30 in the present study) and degree of emphysema (e.g., %LAA = 29% in [3] versus 11% in the present study). Further, we observed that FFMI was independently associated with level of emphysema. This finding strengthens the hypothesis of defective regeneration processes of soft tissue in subjects with COPD and the emphysema phenotype. Although the reason for this is not yet known, various hypotheses are brought to mind. Emphysema is characterized by enlargement of the distal airspaces, caused by destruction of the airway walls [17]. It is postulated that the inflammatory response which results in protease antiprotease imbalance, and therefore tissue destruction is extended from the lungs to whole body soft tissue. However, the link between the respiratory and the systemic inflammatory processes has to be proven yet. Another hypothesis purports emphysema as an autoimmune disease affecting the lung and other systemic elastin-rich tissues in emphysema patients [18]. The aforementioned hypotheses are based on the assumption that loss of soft tissue is a consequence of emphysema. Indeed, patients with severe emphysema undergoing lung volume reduction surgery showed an increase in fat free mass after 6 months, implying that the systemic effects of the disease can, at least partly, be reversed [19]. There are however no convincing data to confirm that muscle wasting is secondary to emphysema yet. In fact, it has been shown that anorexia patients have lower CT density when compared to healthy subjects [20], pointing to the hypothesis that starvation *per se* can induce secondary emphysema. In addition, although most subjects with muscle wasting had high emphysema scores, the predictive value of FFMI for emphysema is only moderate, suggesting that various pathways are involved in the pathogenesis of emphysema. More research is warranted to unravel the temporal sequence of muscle wasting, the presence of emphysema phenotype, and the interrelation between the two. 

In the current study, FMI is a positive covariate of AWT-Pi10 in both subjects with and without COPD. Results of previous studies concerning the correlation between airway wall thickness and BMI are controversial; studies have shown no [2] and positive correlations [5]. Until now, no study related airway wall thickness with FMI. The positive association between the two seen in the present study strengthens the hypothesis of the bronchitis phenotype as CT measured airways were associated with symptoms of chronic bronchitis [15]. However, the association is not disease-specific, questioning the usefulness of AWT-Pi10 as a specific marker to phenotype COPD. 

The association between the CT phenotypes and body composition parameters did not differ between men and women. We have recently shown that there is a gender specific difference in both %LAA and AWT-Pi10 [7]. Taken together, these findings imply that men are more susceptible to develop emphysema and thicker airway walls while the extrapulmonary manifestations of the COPD phenotypes are likely gender independent, at least for body composition.

We would like to address some limitations and strengths of the study. First, the subjects without COPD were younger than the subjects with COPD, and even tough age has been taken into account in the multivariate regression analysis, it could still influence our findings. Secondly, while COPD is considered to be a small airways disease and we calculated AWT-Pi10 using larger airways (Pi > 0.6 cm), studies have shown that CT estimates of airway wall thickness are correlated with histological measurements of small airways [21]. Finally, obese subjects have more subcutaneous fat through which the X-rays have to penetrate resulting in less X-ray energy detected by the CT scanners and X-ray “beam hardening” effects resulting in differential effects on X-ray attenuation values. The increased amount of abdominal fat in obese subjects will also, in the supine position used during CT assessment, tend to decrease the measured lung volume. Both these factors will tend to obscure the observed relationship between %LAA and BMI. However, in the subjects without COPD, no correlation was found between %LAA and BMI, implying that the impact of this technical issue is minimal. The strengths of the present study are the large number of subjects of both genders with and without COPD, and the quantitative analysis of the CT scan. 

To conclude, the present study strengthens the concept that COPD cases with muscle wasting are mainly characterized by the emphysema phenotype and decreased lung mass, although muscle wasting is not discriminative for emphysema. Airway wall thickness is positively related to fat mass index in both subjects with and without COPD. Whether airway wall thickness is a specific marker useful in phenotyping COPD has to be further investigated. These findings provide new insights into the systemic patho-physiology of COPD and its phenotypes. 

## Figures and Tables

**Table 1:** General characteristics of the study subjects stratified for gender.

	Subjects without COPD		Subjects with COPD	
	Men	Women	Men	Women
No. of subjects, *n *	238	221	299	164
Age, y	55.9 ± 9.6	54.8 ± 9.1	65.2 ± 9.4*	62.6 ± 9.0^∗,†^
FEV1, %pred	93.9 ± 8.4	95.9 ± 9.4^†^	52.6 ± 17.4*	53.1 ± 16.1*
FEV1/FVC	0.79 ± 0.04	0.79 ± 0.04	0.52 ± 0.13*	0.54 ± 0.12^∗,†^
Weight, kg	86.0 ± 13.1	70.9 ± 12.8^†^	79.5 ± 14.9*	66.3 ± 16.0^∗,†^
BMI, kg/m^2^	27.0 ± 3.4	25.8 ± 4.5^†^	25.8 ± 4.2*	24.9 ± 5.6
FFMI, kg/m^2^	19.3 ± 1.6	16.8 ± 1.7^†^	18.7 ± 1.6*	15.8 ± 1.9^∗,†^
FMI, kg/m^2^	7.7 ± 2.6	8.9 ± 3.5^†^	7.1 ± 2.8*	9.1 ± 3.9^†^
Lung mass, g	905.2 ± 138.9	753.0 ±113.5^†^	875.0 ± 134.5*	716.9 ± 109.2^∗,†^
No. of pack years, y	21.2 ± 14.4	17.2 ± 12.6^†^	33.9 ± 18.6*	25.5 ± 14.2^∗,†^
AWT-Pi10, cm	0.49 ± 0.03	0.46 ± 0.03^†^	0.50 ± 0.03*	0.47 ± 0.03^∗,†^
%LAA^(log transformed)^	1.04 ± 1.05	0.72 ± 0.89	12.43 ± 11.78*	9.94 ± 11.66^∗,†^

Data are mean ± SD. FEV1: forced expiratory volume in one second, FEV1/FVC: tiffeneau index, BMI: body mass index, FFMI: fat-free mass index, FMI: fat mass index, AWT-Pi10: standardized airway wall thickness at an internal perimeter of 10 mm, %LAA: percentage low attenuation areas less than −950 HU. *P < .05  versus subjects without COPD, ^†^ *P* < .05  versus male counterparts.

**Table 2:** Spearman correlation coefficient between %LAA and AWT-Pi10 and markers of body composition.

	Men		Women	
	%LAA	AWT-Pi10	%LAA	AWT-Pi10
*Subjects without COPD*				
BMI	0.03	0.41^†^	−0.10	0.32^†^
FFMI	−0.02	0.23^†^	−0.05	0.25^†^
FMI	0.03	0.42^†^	−0.10	0.27^†^

*Subjects with COPD*				
BMI	−0.40^†^	0.33^†^	−0.34^†^	0.34^†^
FFMI	−0.43^†^	0.29^†^	−0.39^†^	0.37^†^
FMI	−0.34^†^	0.31^†^	−0.30^†^	0.32^†^

BMI: body mass index, FFMI: fat-free mass index, FMI: fat mass index, AWT-Pi10: standardized airway wall thickness at an internal perimeter of 10 mm, %LAA: percentage low attenuation areas less than −950 HU. Significant correlation: *P < .05, ^†^ *P* < .01.

**Table 3:** Multiple linear regression analyses of change in emphysema level (%LAA) and change in airway wall thickness (AWT-Pi10) by level fat-free mass index (FFMI) and level of fat mass index (FMI).

	Log(%LAA)*			AWT-Pi10**		
	Beta	SE	*P *value	Beta	SE	*P*-value
*Subjects without COPD*						
FFMI	−0.04	0.02	.528	0.16	0.01	.002
FMI	0.09	0.01	.053	0.22	0.01	<.001
*Adjusted R^2^*	*0.26*			*0.33*		

*Subjects with COPD*						
FFMI	−0.28	0.02	.002	0.08	0.02	.460
FMI	0.03	0.01	.686	0.20	0.01	.020
*Adjusted R^2^*	*0.48*			*0.30*		

%LAA: percentage low attenuation areas less than −950 HU, AWT-Pi10: standardized airway wall thickness at an internal perimeter of 10 mm, FFMI: fat-free mass index, FMI: fat mass index.

*Adjusted for age, pack year, FEV1 (%pred), and AWT-Pi10,

**Adjusted for age, pack year, FEV1 (%pred), and %LAA.
